# The METOD study: exploring metabolism, emotional blunting and treatment outcomes in depression – A naturalistic, two-phase observational protocol

**DOI:** 10.3389/fphar.2026.1770558

**Published:** 2026-06-17

**Authors:** Anna Julia Krupa, Michał Jan Kania, Karolina Podkowa, Eve Krawczyk, Iga Plencler-Turakiewicz, Marcin Siwek

**Affiliations:** 1 Department of Biological and Community Psychiatry, Jagiellonian University Medical College, Cracow, Poland; 2 Clinical Department of Adult, Child and Adolescent Psychiatry, University Hospital, Cracow, Poland; 3 Department of Pathophysiology, Jagiellonian University Medical College, Cracow, Poland

**Keywords:** ADHD, chronotype, emotional blunting, insulin resistance, MDD, PTSD

## Abstract

**Introduction:**

Major depressive disorder (MDD) constitutes a substantial global health burden, profoundly undermining psychosocial functioning and quality of life, and the persistent limitations of treatment efficacy—despite advances in neurobiology and pharmacotherapy—underscore its considerable clinical complexity. Recent research increasingly delineates the heterogeneity of depressive symptomatology, particularly emotional blunting, and emerging evidence further implicates metabolic and inflammatory pathways processes in shaping treatment response**.** Insulin resistance (IR), immune dysregulation, individual circadian preference, and trait-level vulnerabilities associated with neurodevelopmental or trauma-related characteristics may contribute to reduced responsiveness to standard therapies and limited adherence to antidepressant treatment. Taken together, these converging findings emphasize the need for individualized interventions rather than continued reliance on broad diagnostic categories.

**Objectives:**

The study consists of two phases with distinct hypotheses: phase I) evaluating emotional blunting along other clinical/psychopathological variables as potential predictors of treatment non-response to SSRI/SNRI in MDD, and phase II) assessing relationships between IR and treatment non-response to vortioxetine in MDD. Further exploratory objectives encompass assessment of: links between psychopathological and metabolic predictors of health status; longitudinal association between emotional blunting and both sexual and cognitive functioning.

**Materials and Methods:**

The METOD study is an open, non-randomized two-phase observational study conducted in accordance with clinical standards and with all required ethical approvals. The protocol was prepared in line with STROBE recommendations. In Phase I, MDD patients meeting eligibility criteria and initiating antidepressant treatment with one of the first-line SSRIs or SNRIs are observed, whereas in Phase II, individuals showing an inadequate response are switched to vortioxetine as a second-line option. Validated clinical assessment tools are administered alongside anthropometric and laboratory evaluations.

**Expected Implications/Significance:**

By examining these interrelations within a naturalistic cohort treated in accordance with current clinical standards, this innovative study—with its strong emphasis on patient-reported outcomes—may increase insight into the lived experience of depression, enhance understanding of the pathophysiological mechanisms shaping its diverse symptomatology, and provide a foundation for more precisely targeted therapeutic approaches.

## Introduction

### Background and rationale

Major depressive disorder (MDD) is widely recognized as one of the most formidable global public health challenges, given its profound impact on psychosocial functioning, family dynamics, and the substantial economic burden it imposes (Papola et al., 2024).

Despite advances in research on the neurobiological mechanisms of MDD and the development of pharmacotherapy, the overall clinical effectiveness of treatment remains unsatisfactory, a fact that continues to be a source of considerable scientific debate and controversy (Moncrieff, 2018). In fact, presently available antidepressant medications, including selective serotonin reuptake inhibitors (SSRIs) and serotonin–norepinephrine reuptake inhibitors (SNRIs), demonstrate efficacy, but the response and remission rates remain unsatisfactory (Cipriani et al., 2018). In the initial report of the Sequenced Treatment Alternatives to Relieve Depression (STAR*D) trial, the remission rate after the first step, which involved the use of the SSRI citalopram, was approximately one-third of patients, and with successive, escalated treatment steps, the cumulative remission rate was reported to reach about 67% (Sinyor et al., 2010). However, more recent reanalyses of these data, conducted in strict accordance with the study’s original protocol, indicate that the actual cumulative remission rate was considerably lower—approximately half of the initially reported value (Pigott et al., 2023). This modest effectiveness may, in part, be explained by the fact that depression is diagnosed solely based on non-specific symptom classifications, whereas clinically valuable and precise biomarkers capable of stratifying a heterogeneous patient population, predicting therapeutic response, and enabling treatment personalization are still scarce (Buch and Liston, 2021).

At the same time, reliably assessing symptom improvement remains challenging, thereby reinforcing the shift toward patient-reported outcomes that capture the subjective dimensions of quality of life not always adequately reflected by clinician-rated measures (Correll et al., 2025; Krupa, 2026). Consequently, greater emphasis is placed on achieving functional remission, defined as the restoration of social and occupational functioning, surpassing the traditional focus on symptomatic remission alone (Yang et al., 2022).

Moreover, it has been proposed that specific symptom phenotypes (e.g., core depression, anxiety, neurovegetative symptoms of melancholia, atypical depression) should be distinguished, as these may delineate distinct clinical manifestations of depression with differential treatment responsiveness (Ahmed et al., 2018; Bayes and Parker, 2019).

In line with this multidimensional understanding of MDD symptomatology, recent research has increasingly focused on emotional blunting, a phenomenon that significantly affects quality of life—particularly in terms of energy, motivation, and psychosocial functioning—and may represent a distinct symptomatic dimension of MDD as well as an adverse effect associated with antidepressant treatment, particularly with SSRIs and SNRIs (Ma et al., 2021). Furthermore, emotional blunting has been reported to be associated with SSRI-induced sexual dysfunction, but this relationship has not been systematically investigated in prospective studies or among patients receiving antidepressants with other mechanisms of action (Opbroek et al., 2002). Although evidence suggests that individuals with a history of trauma tend to exhibit higher levels of emotional blunting, research examining the associations between post-traumatic stress symptoms and the severity of emotional blunting in depression remains scarce (Christensen et al., 2022b). Interestingly, vortioxetine, characterized by a unique mechanism of action as a serotonin modulator and stimulator, appears effective in reducing emotional blunting in patients with MDD who exhibit significantly reduced emotional responsiveness and have an inadequate response to SSRIs/SNRIs treatment (Fagiolini et al., 2021; Sampson et al., 2024). Therefore, ongoing research efforts aimed at identifying objective measures to navigate the personalization of antidepressant therapy have highlighted several mechanistic domains, such as metabolic dysregulation, inflammation-related processes, and disturbances within the hypothalamic–pituitary–adrenal (HPA) axis (Lamers et al., 2013). Recent evidence indicates that alterations within the former two domains, namely, metabolic and inflammatory dysregulation, may characterize up to 30% of individuals with depression (Penninx et al., 2025).

Particular attention has been drawn to parameters related to metabolic dysregulation, as emerging evidence suggests that it is significantly associated with the clinical features of depression, including links between insulin resistance (IR) and changes in appetite and weight, anhedonia, cognitive impairments, irritability, fatigue, and hypersomnia (Brouwer et al., 2021; Rashidian et al., 2021; Sen et al., 2021; Krupa, 2025a). Notably, our recent work indicated that IR was a significant predictor of increased anhedonia severity, a core and enduring symptom of MDD (Siwek et al., 2024). Our previous findings from a cross-sectional study showed that higher IR may be associated with reduced responsiveness to SNRIs’ effectiveness in MDD patients (Krupa et al., 2024a, 2024b). Similar results were reported earlier in a *post hoc* analysis of a prospective trial in vortioxetine-treated patients by Rashidian et al., where IR was associated with poorer therapeutic outcomes; however, the inclusion of both diabetic and non-diabetic individuals limits the generalizability of these findings (Rashidian et al., 2021). Vortioxetine has also been associated with poorer treatment outcomes among patients with higher baseline C-reactive protein (CRP) levels (Stahl, 2015; Rashidian et al., 2022; Krupa A. J. et al., 2023). Moreover, elevated CRP at treatment initiation appeared to promote an increase in IR during therapy, which may in turn have contributed to the lack of therapeutic response (Rashidian et al., 2022). Of note, the association between the earlier described emotional blunting and metabolic parameters, particularly IR, has not been systematically examined to date.

Additionally, accumulating evidence suggests that factors such as chronotype—particularly an evening chronotype—as well as attention-deficit/hyperactivity disorder (ADHD) symptoms and post-traumatic stress disorder (PTSD) may influence the course of MDD and potentially affect antidepressant treatment outcomes (Chen et al., 2016; Taylor and Hasler, 2018; Angelakis et al., 2020; Mao et al., 2024; Krupa, 2025b). To date, only a few studies, including a pilot analysis conducted by members of our research team, have suggested that an evening chronotype may be a potential predictor of inadequate response to pharmacotherapy with SSRIs or SNRIs in depression (McGlashan et al., 2018; Krupa A. et al., 2023). While current evidence is limited, existing studies suggest that comorbid ADHD may increase the risk of treatment-resistant MDD, yet it remains uncertain whether this association is consistent across different classes of antidepressant agents or specific to certain pharmacological mechanisms of action (Chen et al., 2016; Sternat et al., 2018). Integrating chronobiological, post-traumatic, and ADHD symptom domains into future research may provide the foundation for a more precise and evidence-based approach to MDD management, enabling more accurate diagnosis, improved prediction of treatment response, and the development of interventions tailored to individual patients (Fernandes et al., 2017).

### Objectives

This work builds on the concept that specific clinical features of MDD—particularly symptomatic dimension of emotional blunting, an evening chronotype, the presence of clinically significant ADHD and PTSD symptoms metabolic factors (notably IR) may be associated with treatment response following SSRIs, SNRIs, and vortioxetine therapy. This study aims to elucidate these relationships under real-world clinical conditions in the naturalistic cohort of patients treated in accordance with established therapeutic standards.

The METOD study consists of two sequential phases with distinct hypotheses: Phase I focuses on clinical and psychopathological predictors, evaluating emotional blunting as a potential predictor of treatment non-response to SSRI/SNRI in MDD, and Phase II will assess if IR is linked to treatment non-response to vortioxetine in MDD. Further exploratory objectives of the METOD study consist of an assessment of links between psychopathological and metabolic predictors of health status; and evaluation of longitudinal association between emotional blunting and sexual as well as cognitive functioning.

The anticipated findings are expected to deepen the understanding of the pathophysiological mechanisms underlying MDD and inform the development of more individualized therapeutic approaches.

## Materials and methods

### Study design

The METOD study: exploring Metabolism, Emotional blunting and Treatment Outcomes in Depression is a non-randomized, open-label, observational study designed to analyze the psychopathological and metabolic factors influencing the effectiveness of unipolar depression treatment. The study does not entail any experimental interventions. All participants will receive treatment in accordance with current therapeutic standards of MDD (Samochowiec et al., 2021). Enrollment or discontinuation will have no influence on the course of treatment or on clinical decisions made by the attending physicians. The METOD study protocol was constructed in line with STROBE recommendations (Supplementary Material).

The study will use a broad set of standardized psychometric instruments assessing depressive symptom severity, overall clinical status, trauma exposure and post-traumatic symptomatology, sexual functioning, sleep quality, health-related quality of life, resilience, circadian preferences, cognitive performance, attention deficit symptoms, and nicotine dependence (Table 1). Both clinician-rated and self-report measures will be used, complemented by anthropometric assessments and biochemical analyses.

**TABLE 1:** Clinical tools and assessment schedule.

​	​	​	Time points (weeks)					
			Phase I			Phase II		
Type of outcomes	Tool	Construct measured	Baseline	2 week	8 week	Baseline	2 week	8 week
Clinician-reported	CGI	Global clinical evaluation	●	●	●	●	●	●
	MADRS	Depressive symptom severity	●	●	●	●	●	●
	FTND	Severity of nicotine dependence	●	​	​	●	​	​
Patient-reported	AIS	Sleep quality	●	●	●	●	●	●
	ASEX	Sexual functioning	●	●	●	●	●	●
	GAD-7	Anxiety symptoms	●	●	●	●	●	●
	ITQ	Trauma-related symptoms	​	​	●	​	​	​
	ITEM	Exposure to traumatic events	​	●	​	​	​	​
	ODQ	Depressive symptoms, including emotional blunting	●	●	●	●	●	●
	QIDS	Depressive symptoms	●	●	●	●	●	●
	EQ-5D-5L	Overall quality of life and health	●	●	●	●	●	●
	BRCS	Resilience	●	●	●	●	●	●
	CSM	Chronotype	●	​	​	​	​	​
	THINC-it	Cognitive dysfunction in adults with MDD	​	​	​	●	​	●
	ASRS	ADHD symptoms in adults	●	​	​	​	​	​
Anthropometric measures	​	Height, body weight, WHR	●	●	●	●	●	●
Laboratory tests	Blood samples	CBC, CRP, lipid profile, HOMA-IR	​	​	​	●	​	●

**AIS**, Athens Insomnia Scale; **ASEX**, Arizona Sexual Experiences Scale; **ASRS**, Adult ADHD, Self-Report Scale; **BRCS**, Brief Resilience Coping Scale; **CBC**, Complete Blood Count; **CGI**, Clinical Global Impression Scale; **CRP**—C‐Reactive Protein; **CSM**, Composite Scale of Morningness; **EQ-5D-5L**—EuroQol 5-Dimension 5-Level Questionnaire; **FTND**, Fagerström Test for Nicotine Dependence; **GAD-7**, Generalized Anxiety Disorder-7 scale; **HOMA-IR**, Homeostasis Model Assessment of Insulin Resistance; **ITEM**, International Trauma Exposure Measure; **ITQ**, International Trauma Questionnaire; **MADRS**, Montgomery–Åsberg Depression Rating Scale; **ODQ**, Oxford Depression Questionnaire; **QIDS**, Quick Inventory of Depressive Symptomatology; **WHR**, Waist-to-Hip Ratio (Guy, 1976; Fagerström, 1978; Montgomery and Asberg, 1979; Smith et al., 1989; McGahuey et al., 2000; Rush et al., 2003; Sinclair and Wallston, 2004; Kessler et al., 2005; Spitzer et al., 2006; Busner and Targum, 2007; Herdman et al., 2011; Price et al., 2012; McIntyre et al., 2017; Cloitre et al., 2018; Hyland et al., 2021).

The recruitment started in February 2025 and will continue until December 2028. Participants are recruited from both outpatient and inpatient psychiatric settings in Cracow, Poland.

**FIGURE 1:**
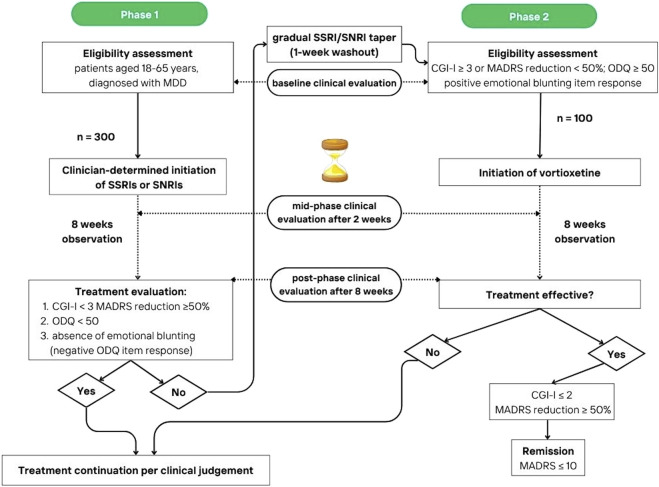
Study flowchart.

The study comprises two sequential phases (Figure 1). Study visits will be scheduled at weeks 0 (baseline), 2, and 8 in both phases. During these visits clinical and self-reported parameters will be assessed according to the measurement framework presented in Table 1. Phase I will include patients aged 18–65 years, either hospitalized or treated in an outpatient setting, who meet the diagnosis of a first episode of MDD or MDD in the course of recurrent depression according to the DSM-5 (Diagnostic and Statistical Manual of Mental Disorders, Fifth Edition). Patients will be eligible for inclusion on the condition that they have not received prior pharmacological treatment for the current depressive episode, which must not exceed a duration of 12 months. Participants who achieve a satisfactory treatment response will complete their involvement after the 8-week observation period of Phase I. Later their clinical care will continue in accordance with current clinical guidelines for MDD. Patients who fail to achieve an adequate response after at least 6 weeks of monotherapy with an SSRI or SNRI, defined by a score of ≥3 on the Clinical Global Impression–Improvement (CGI-I) scale or a reduction of <50% on the Montgomery–Åsberg Depression Rating Scale (MADRS), and who exhibit clinically significant emotional blunting, indicated by a total score of ≥50 on the Oxford Depression Questionnaire (ODQ), will be invited to Phase II, in which they will be switched to vortioxetine therapy (Table 2), (Guy, 1976; Montgomery and Asberg, 1979; Busner and Targum, 2007; Price et al., 2012). The cut-off score ≥50 on the ODQ was defined based on earlier studies in MDD subjects, determining it as a threshold for substantial degree of emotional blunting (Christensen et al., 2021; Fagiolini et al., 2021). Short-term adjunctive treatments such as benzodiazepines, trazodone, quetiapine or pregabalin will be permitted. At the 8-week endpoint of Phase II, patients will complete their participation in the study, and their clinical care will continue in accordance with current clinical guidelines for MDD management.

**TABLE 2:** Therapeutic strategies in Phase I and Phase II of the study.

Phase I		Range of doses (mg/24 h)
SRI	Citalopram	20–40
	Escitalopram	10–20
	Fluoxetine	20–60
	Fluvoxamine	150–300
	Paroxetine	20–50
	Sertraline	50–200
SNRI	Duloxetine	30–120
	Venlafaxine	75–225

Phase II		
Other	Vortioxetine	10–20

The planned self-report instruments are expected to take 30 min (±5min) to complete upon the first visit of the I Phase and 20 min (±5min) to complete upon the following visits. The planned clinician-rated evaluations are expected to take 20 min (±5min), with the exception of visits I and III in Phase II which are expected to take 30 min (±5min). The assessment schedule has already been piloted in 23 patients, yielding a high compliance: no complaints were noted on the recruited patients assessing clinicians part.

The study is conducted in full compliance with internationally accepted ethical standards for clinical research and applicable patient safety regulations. Written informed consent will be obtained from each participant before enrollment. All collected data will be encoded and stored within a secure, access-restricted database.

### Evaluation of pragmatism

To assess the degree of pragmatism in the study design, the Pragmatic–Explanatory Continuum Indicator Summary-2 (PRECIS-2) framework was applied, in which each of nine domains is rated on a five-point scale ranging from 1 (highly explanatory) to 5 (highly pragmatic) (Loudon et al., 2015). This tool evaluates how closely a study reflects real-world clinical practice by examining the following dimensions: eligibility criteria, recruitment, setting, organization, flexibility in delivery and adherence, follow-up, primary outcomes, and analysis. Initially designed for randomized controlled trials (RCTs), the PRECIS-2 framework can be adapted for observational, non-randomized studies to systematically assess their pragmatic features and enhance transparency regarding their clinical relevance and applicability.

The METOD study was conceived to emulate real-world clinical conditions as faithfully as possible.

The inclusion criteria were deliberately broad, and patient recruitment took place within the context of standard outpatient and inpatient psychiatric care. All therapeutic decisions, including dose titration, adjunctive treatments, and the continuation or discontinuation of pharmacotherapy, were left entirely to the discretion of the treating physician. Research procedures were limited to indispensable clinical, psychometric, and laboratory assessments, while the assessment schedule was aligned with patients’ routine follow-up visits. Such a design maximizes external validity and enhances the generalizability and applicability of the results to everyday psychiatric practice. The METOD study achieved high scores across most PRECIS-2 domains, indicating a predominantly pragmatic design. The mean domain score was 3.8 (range: 3–5). The overall results with detailed ratings for each domain are presented in Table 3.

**TABLE 3:** PRECIS-2 domain ratings for the METOD study adapted from (Loudon et al., 2015).

Eligibility criteria (3/5)
The inclusion criteria for phase I of the study encompass a broad, representative patient population, while exclusions are limited to severe psychiatric or somatic comorbidities, supporting external validity and a pragmatic study design. The inclusion criteria for phase II of the study encompass a more defined population, which receives a particular drug (vortioxetine), but remains representative for a large population of patients who failed to adequately respond to an SSRI/SNRI.
Recruitment (4/5)
Participants are continuously enrolled in routine inpatient and outpatient psychiatric care, reflecting everyday clinical practice.
Setting (4/5)
The study is conducted in two standard psychiatric facilities, encompassing both outpatient and inpatient settings, without additional organizational requirements and integrated into routine psychiatric care.
Organization (4/5)
Research procedures are integrated into routine clinical practice (follow-up visits, laboratory tests, clinical assessments), with only minimal additional questionnaire-based measurements introduced.
Flexibility in delivery (5/5)
Therapeutic decisions, including dosage adjustments, temporary adjunctive interventions, and treatment continuation or discontinuation, were made solely at the discretion of the treating psychiatrist, reflecting routine psychiatric practice.
Flexibility in adherence (4/5)
Treatment adherence was monitored through standard clinical and self-report measures, with no additional enforcement mechanisms. This limited oversight reflects a pragmatic approach, though slightly less flexible than fully naturalistic conditions.
Follow-up (4/5)
Follow-up visits were scheduled at baseline, week 2, and week 8, consistent with common clinical practice. Although slightly more structured than typical care, the schedule imposed minimal additional burden on participants.
Primary outcomes (3/5)
Although clinically relevant, the primary and secondary outcomes require specialized assessments beyond routine clinical practice, thereby introducing explanatory features into the otherwise pragmatic study design.
Primary analysis (3/5)
The statistical analysis plan employed standard comparative and correlational methods appropriate to an observational design, rendering this domain more explanatory than fully pragmatic.

### Eligibility criteria and study design

The following inclusion and exclusion criteria will be applied. Participation in the study will be possible only after obtaining signed informed consent from the subject.

#### Phase I

##### Inclusion criteria

- Female and male participants aged 18–65 years;
- Diagnosis of depression according to DSM-5 criteria;
- Duration of the current depressive episode ≤12 months;
- No prior pharmacological treatment for the current depressive episode.

##### Exclusion criteria

- Prisoners and individuals treated involuntarily due to psychiatric or somatic illness;
- Any other psychiatric diagnoses (e.g., bipolar disorder, schizophrenia, schizoaffective disorder, substance dependence excluding nicotine and caffeine);
- Severe, acute, or chronic somatic or neurological diseases, including diabetes, and clinically significant neurological disorders such as epilepsy, Parkinson’s disease, Alzheimer’s disease, multiple sclerosis, history of stroke, transient ischemic attacks, cerebral palsy, or intellectual disability;
- Pregnancy or breastfeeding;
- Concomitant use of drugs inducing antidepressant metabolism (e.g., rifampicin, glucocorticosteroids, phenytoin);
- Previous antidepressant treatment during the current episode.

#### Phase II

##### Inclusion criteria

- Completion of Phase I of the study;
- History of SSRI (sertraline, citalopram, escitalopram, paroxetine, fluoxetine, fluvoxamine) or SNRI (duloxetine, venlafaxine, milnacipran) monotherapy for the current depressive episode at an adequate dose for ≥6 weeks;
- Inadequate response to SSRI/SNRI treatment, defined as CGI-I score ≥3;
- Clinically significant emotional blunting, defined as ODQ ≥ 50 and a positive response to a qualifying question assessing emotional numbness, detachment, or reduced emotional responsiveness during the past 6 weeks (Price et al., 2012; Christensen et al., 2022a).

##### Exclusion criteria

- a–e. As specified in the Phase I exclusion criteria;
- Nonresponse to ≥2 adequate antidepressant treatment trials (minimum 6 weeks at therapeutic doses) during the current depressive episode.
- Patients whose clinical condition would require a drug other than vortioxetine will be excluded from Phase II of the study.

### Interventions

Phase I consists of a prospective observation of patients with MDD treated with one of the SSRIs—sertraline, citalopram, escitalopram, paroxetine, fluvoxamine, or SNRIs—duloxetine or venlafaxine (Table 2). Phase II involves a prospective, longitudinal observation of patients who are switched from an SSRI or SNRI to vortioxetine due to an inadequate response to prior antidepressant therapy. All medications used in the study are approved for the treatment of depressive episodes and are routinely prescribed in standard clinical practice. The choice of antidepressant in Phase I and the selection of doses across both phases are flexible and determined by the treating clinician, based on a comprehensive evaluation of the patient’s mental status, psychiatric and somatic comorbidities, and potential contraindications or drug–drug interactions.

### Statistical analysis

All statistical analyses will be conducted using the R software (R Core Team, 2025). Descriptive statistics will be employed to summarize the data. Qualitative variables will be presented as frequencies and percentages, and continuous variables as means with standard deviations or medians with interquartile ranges, as appropriate. Preliminary analyses will consist of contingency tables and Chi-square tests for categorical variables, and analysis of variance (ANOVA) or non-parametric equivalents for continuous variables. Moreover, multivariable regression analyses will be performed to examine whether metabolic variables and emotional blunting are predictive of depression treatment outcomes. A two-tailed p-value < 0.05 will be considered statistically significant.

Sample size calculations were performed to determine the minimal number of participants that would allow for the study to detect clinically significant difference between treatment response and non-response with 95% power (α = 0.05). Based on available data from the Polish population on IR in MDD patients, the minimal number of participants would be 26 treatment-responsive and 26 treatment non-responsive participants (Krupa et al., 2024b).

Based on available data from MDD clinical trials, a 50% response rate (STAR*D) and 20%–40% attrition were assumed at both Phase I and Phase II (Sinyor et al., 2010). Moreover, it was assumed that most patients will present significant emotional blunting, as it has been shown to affect nearly all patients (over 99%) in acute depression (Christensen et al., 2022b).

The study is planned to enroll 300 participants in Phase I. This results in an anticipated final analytical sample of approximately 90–120 participants in Phase II. Approximately 45–60 treatment responders and 45–60 treatment non-responders are anticipated in Phase II, which exceeds the minimum requirement to differentiate the treatment response and non-response groups based on IR and provides sufficient robustness for multivariable analyses.

Statistical analyses will be conducted separately for each study phase.

### Phase I

Two multivariable logistic regression models will be built with the following dependent variables

1. clinician-reported treatment non-response defined as a reduction of < 50% on the MADRS;
2. patient-reported treatment non-response defined as a <50% reduction on the QIDS-SR.

Candidate covariates will include demographic variables (age, sex), anthropometric measures (waist-to-hip ratio, WHR), continuous psychopathological variables (emotional blunting, insomnia, sexual functioning, anxiety, chronotype, resilience), and binary clinical variables (nicotine dependence, clinically significant symptoms of PTSD, and ADHD).

Additionally, a multivariable linear regression model will be built with health status, as assessed with the EQ-5D-5L, as the dependent variable, and the candidate covariates listed for the multivariable logistic regression models described above.

### Phase II

Given the anticipated sample size, the number of predictors will be limited to ensure model stability. The final models will include IR, an immunometabolic Z-score (including lipid profile, CRP, and waist-to-hip ratio), a cognitive functioning Z-score (based on overall THINC-it results), and baseline depression severity (assessed with MADRS).

Two multivariable logistic regression models will be built with the following dependent variables

1. Clinician‐reported treatment non-response defined as a reduction of < 50% on the MADRS;
2. Patient‐reported treatment non-response defined as a <50% reduction on the QIDS-SR.

A multivariable linear regression model will be built using health status, as assessed with the EQ-5D-5L, as the dependent variable, with the candidate covariates listed for the multivariable logistic regression models described above.

Additional exploratory longitudinal analyses will be conducted using mixed-effects models (MEM) to explore the longitudinal relationship between baseline emotional blunting (continuous variable) and sexual functioning (as evaluated with ASEX) and cognitive Z-score (as evaluated by THINC-it), which will both be included as continuous variables.

These analyses are considered exploratory and hypothesis-generating. Hence, their results will be interpreted accordingly, without formal adjustment for multiple comparisons.

In Phase I, the longitudinal association between baseline emotional blunting and sexual functioning will be assessed across all three time points. In Phase II, the longitudinal association between baseline emotional blunting and cognitive functioning (expressed as a Z-score) will be evaluated across all three time points. For these analyses, MEM will be applied, including time, baseline emotional blunting, and their interaction as fixed effects, with a random intercept for participants.

Missing data will be handled using a complete-case approach. To assess potential bias, baseline characteristics of participants with complete data will be compared with those of participants with missing data. Sensitivity analyses will be performed to evaluate the robustness of the findings. These will include: alternative definitions of treatment outcome (remission-based thresholds, with clinician-rated remission defined as MADRS score <10 and patient-rated remission defined as QIDS-SR score <6 at the third visit in both study phases) and comparison of full and parsimonious regression models.

## Ethical statement

The METOD study is conducted in accordance with the principles of research ethics, the Declaration of Helsinki, and local institutional approval from the Bioethics Committee of the Jagiellonian University in Cracow, Poland (approval no. 1072.6120.117.2024), with particular attention to the wellbeing and rights of participants (World Medical Association, 2025).

The study is expected to provide value at both individual and population levels through a comprehensive assessment of mental health status and access to relevant laboratory results. For participants, this will entail an in-depth understanding of the nature and determinants of their symptoms, as well as a thorough evaluation of overall health to facilitate optimization of ongoing treatment. Participants will receive detailed feedback on the results of the performed assessments to promote their self-awareness and active participation in the treatment process.

## Outcomes

The study consists of two sequential phases with distinct analytical objectives:

Phase I focuses on clinical and psychopathological predictors, testing the hypothesis that (H1) treatment non-response to SSRIs/SNRIs in MDD is associated with emotional blunting.

Phase II incorporates metabolic assessments, including IR, testing the hypothesis that (H2) treatment non-response to vortioxetine in MDD is associated with IR.

In both Phase I and Phase II, the primary endpoint will be the clinician-rated treatment non-response (defined as a reduction of < 50% in MADRS score), while the key secondary endpoint will be the patient-rated treatment non-response (defined as a reduction of < 50% in QIDS-SR score) as measured at the third visit of each study phase.

Additional analyses in both phases will assume health status (evaluated with EQ-5D-5L) as an exploratory outcome, focusing on either psychopathological (Phase I) or metabolic (Phase II) predictors. Furthermore, exploratory longitudinal analyses will be conducted to assess the relationship between baseline emotional blunting and changes in sexual functioning (Phase I) and cognitive functioning (Phase II).

Table 1 presents the clinical tools and assessment schedules used in the study, selected for their widespread use and established validity in empirical research.

## Discussion

The research project presented in this paper constitutes an advanced endeavor to elucidate the intricate interrelations between the clinical endophenotype of MDD and selected biological and psychological determinants.

In the present study, we utilized well-established and validated clinician-rated measures, namely, the MADRS and the CGI scale. The MADRS is a validated clinician-rated scale for assessing the severity of depressive symptoms, with demonstrated correspondence to the Hamilton Depression Rating Scale (HDRS, HAM-D), that is widely implemented in MDD research (Zimmerman et al., 2004; Kowalczyk et al., 2024). Moreover, validated conversion methods have been established to translate scores between the MADRS and the HAM-D, further supporting the comparability and clinical relevance of both instruments (Leucht et al., 2018). At the same time, the CGI-I scale is widely used as a global measure of clinical change and has been validated against other psychiatric rating scales (Forkmann et al., 2011). Therefore, we believe that the use of the MADRS and CGI in the study provides a robust and clinically meaningful assessment of depressive symptoms and treatment response.

The protocol relies on a seemingly extensive battery of clinician-rated and self-report instruments administered at multiple time points. While this comprehensive approach is a clear strength of the study, it could pose feasibility challenges in routine clinical settings, particularly among acutely depressed patients. However, this has not been our experience with the pilot sample of initially recruited patients. Moreover, some patients have spontaneously expressed contentment in completing standardized assessment instruments, mentioning that they find it difficult to determine which information is important for their treatment and appreciate a more comprehensive clinician’s interest in their mental health.

Incorporation of factors such as emotional blunting, metabolic parameters, chronotype, and comorbid symptoms of ADHD and PTSD facilitates a more precise and individualized evaluation of antidepressant treatment efficacy. This study is both innovative and of substantial scientific relevance, as it emphasizes patient-reported outcomes and thereby contributes to the advancement of personalized therapeutic strategies in depression, while simultaneously enhancing understanding of the pathophysiological mechanisms underpinning MDD symptomatology.

Emotional blunting, one of the most common yet underrecognized adverse effects of treatment, represents a significant clinical challenge due to its potential to contribute to treatment discontinuation among patients (Alcalá et al., 2025). The implementation of vortioxetine in the present study—an agent increasingly used as an alternative to SSRIs/SNRIs—provides an opportunity to adopt a broader analytical perspective and to examine its therapeutic effects in the context of concomitant metabolic and psychiatric comorbidities (Fagiolini et al., 2021; McIntyre et al., 2023; Christensen et al., 2024; d’Andrea et al., 2024).

The inclusion of the HOMA-IR index as a biomarker in the present project may facilitate the identification of a distinct subgroup of patients requiring alternative therapeutic strategies and contribute novel insights to the field, given the considerable heterogeneity characterizing previous findings (Fernandes et al., 2022).

As mentioned above, an evening chronotype, associated with circadian rhythm disruptions, may influence both the severity of depressive symptoms and the effectiveness of antidepressant treatment (McGlashan et al., 2018). Accounting for this factor in the analysis may support the development of more refined therapeutic approaches, enhancing treatment efficacy and clinical outcomes.

Symptoms of ADHD and PTSD, which frequently co-occur with depression, may modulate treatment response and the clinical trajectory of the disorder, with their presence being linked to an increased risk of treatment resistance, thereby warranting systematic assessment within the framework of the project (Chen et al., 2016; Dominguez et al., 2021).

Furthermore, the use of the THINC-it application for cognitive assessment enables an objective evaluation of treatment effects on cognitive functioning, which is frequently impaired in patients with depression yet remains rarely monitored in routine clinical practice (McIntyre et al., 2017). Finally, anthropometric measurements and blood analyses may also provide valuable insights, offering additional objective data relevant to the biological underpinnings of depression and treatment response.

The project is subject to several limitations. Its observational design inherently restricts causal inference, and the absence of randomization should be considered when interpreting the findings. Furthermore, given the specific clinical characteristics of the studied population and the study’s primary objectives, including a conventional healthy control group was not methodologically appropriate (Krupa, 2026). The sole reliance on clinician-reported psychometric instruments introduces potential bias and subjectivity; hence, in this study, both clinician- and patient-rated outcomes were included. Logistical constraints may affect the completeness of laboratory and cognitive data. Additionally, the applied exclusion criteria may limit the external validity of the findings with respect to the broader population of patients with MDD commonly encountered in routine clinical settings. Specifically, exclusions related to severe somatic or neurological comorbidities, concomitant psychiatric diagnoses, and prior antidepressant treatment during the current depressive episode were introduced primarily to safeguard patient safety, reduce potential sources of clinical heterogeneity and confounding, and preserve the interpretability of study outcomes within the methodological constraints of a pragmatic real-world observational design. Although the study assumes a pragmatic approach, the decision to initiate vortioxetine in Phase II is protocol-driven and based on predefined eligibility criteria informed by current literature and clinical rationale. Vortioxetine was selected due to its well-documented efficacy, favorable effects on cognitive function and emotional blunting, as well as evidence from comparative studies indicating advantages over certain alternative second-line treatment options, including agomelatine, in patients with inadequate response to prior treatment with SSRIs or SNRIs (Montgomery et al., 2014). It is worth noting that protocol-driven drug choice is limited to the second phase of the study, excluding the potential bias related to heterogeneity in the effects of antidepressants on metabolic profile and allowing for the evaluation of links between IR and clinical outcomes. The above-mentioned inclusion and exclusion criteria were intended to balance real-world applicability with methodological rigor, ensuring the study’s feasibility while enabling a more reliable evaluation of treatment effectiveness in a clinically relevant population.

In the present study, a healthy control cohort was not included because emotional blunting is not a physiological phenomenon observed in healthy individuals, but rather a clinical feature associated with psychiatric disorders and/or their treatment (Ma et al., 2021). Therefore, a direct comparison with a healthy population would not constitute a meaningful reference for the primary outcomes assessed. Furthermore, the primary objective of this study was to examine the relationship between metabolic parameters and treatment response. In individuals without psychiatric illness, pharmacological treatment is not recommended, which precludes the assessment of treatment response and its association with metabolic variables. Consequently, investigating these relationships in a healthy cohort would be both methodologically inappropriate and clinically uninformative.

Importantly, the study design itself allows for the emergence of clinically relevant control comparisons through the identification of patient subgroups based on treatment outcomes, including responders and non-responders (Krupa et al., 2024b). These naturally occurring subgroups provide an internal comparative framework that is substantially more informative for the study objectives than comparisons with healthy individuals, as they directly reflect variations in treatment response within the target clinical population.

Nevertheless, the findings of this study may provide a robust foundation for the design of future interventional research, in which individual patient characteristics and underlying physiological and psychological factors will serve as key stratification variables. Furthermore, subsequent analyses have the potential to inform the development of predictive algorithms that support evidence-based clinical decision-making in the treatment of depression, in accordance with the principles of precision and personalized medicine.
